# Supplementary material for: The exonic SNP rs11676272-C risk allele mediates diet-induced obesity and reduces enhancer activation
Source: EMBO Rep. 2026 Apr 6;27(9):2462–90. doi: 10.1038/s44319-026-00758-9 (PMC13172560; doi:10.1038/s44319-026-00758-9)
Supplement: Supplementary file 14 — Expanded View Figures [file 44319_2026_758_MOESM14_ESM.pdf]

Expanded View Figures

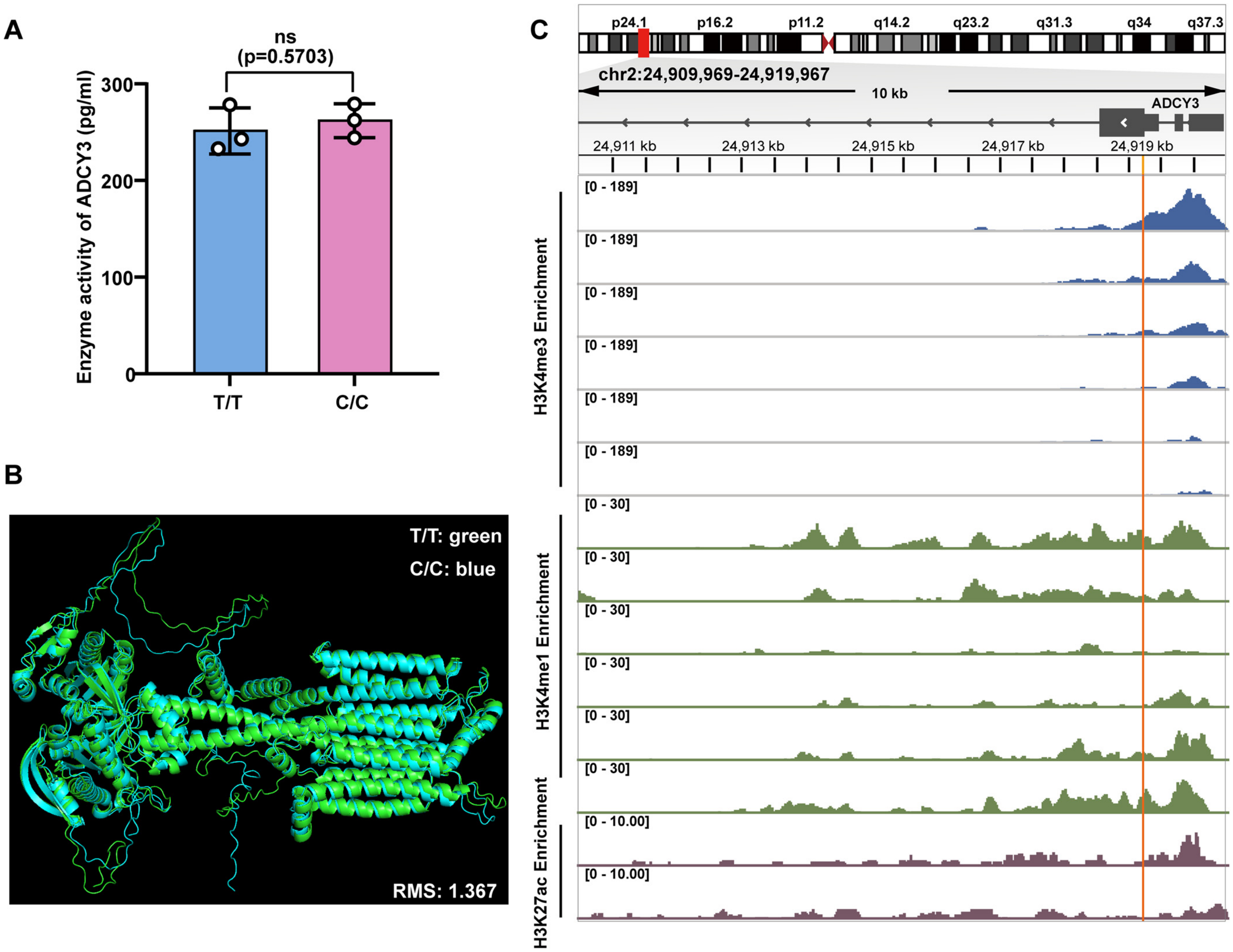

Figure EV1. Characterization of ADCY3 enzymatic activity, protein structure, and locus epigenetics in the context of the rs11676272 variant.

(**A**) ADCY3 enzymatic activity in T/T and C/C cells. $n = 3$ groups from three independent experiments. (**B**) AlphaFold predicted structural models of human ADCY3 carrying rs11676272-T and -C alleles. (**C**) Epigenetic annotations of H3K27ac, H3K4me1, and H3K4me3 marks at the rs11676272 locus in adipose tissue from the ENCODE database. rs11676272 is indicated by the orange vertical line. Data were presented as mean ± SD, ns not significant by Student's *t*-test.

 © The Author(s)

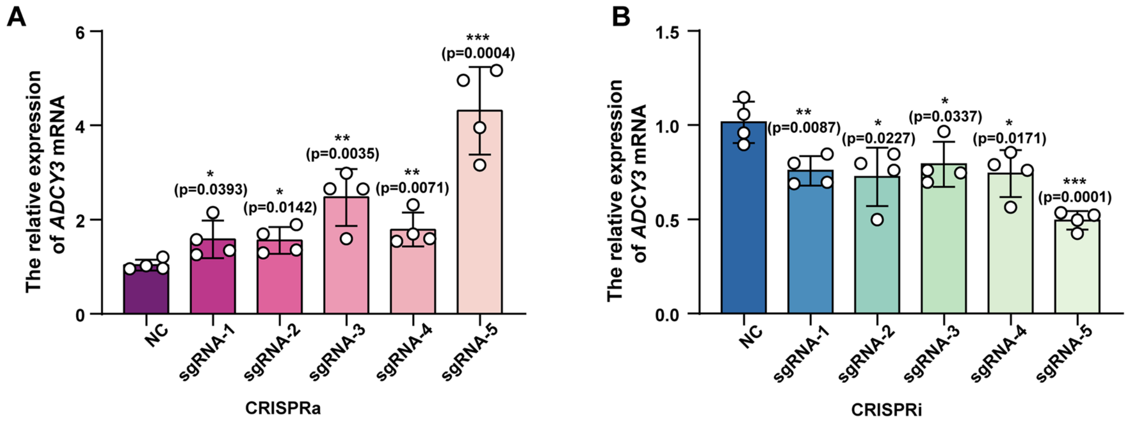

Figure EV2. Validation of the sgRNA efficiency for CRISPR editing.

(**A**) qPCR analysis of *ADCY3* activation using indicated sgRNA sequences. (**B**) qPCR analysis of *ADCY3* inhibition using indicated sgRNA sequences. *GAPDH* was used as internal reference; $n = 4$ groups from three independent experiments; All data were presented as mean ± SD; *$p < 0.05$; **$p < 0.01$, ***$p < 0.001$ by one-way ANOVA.

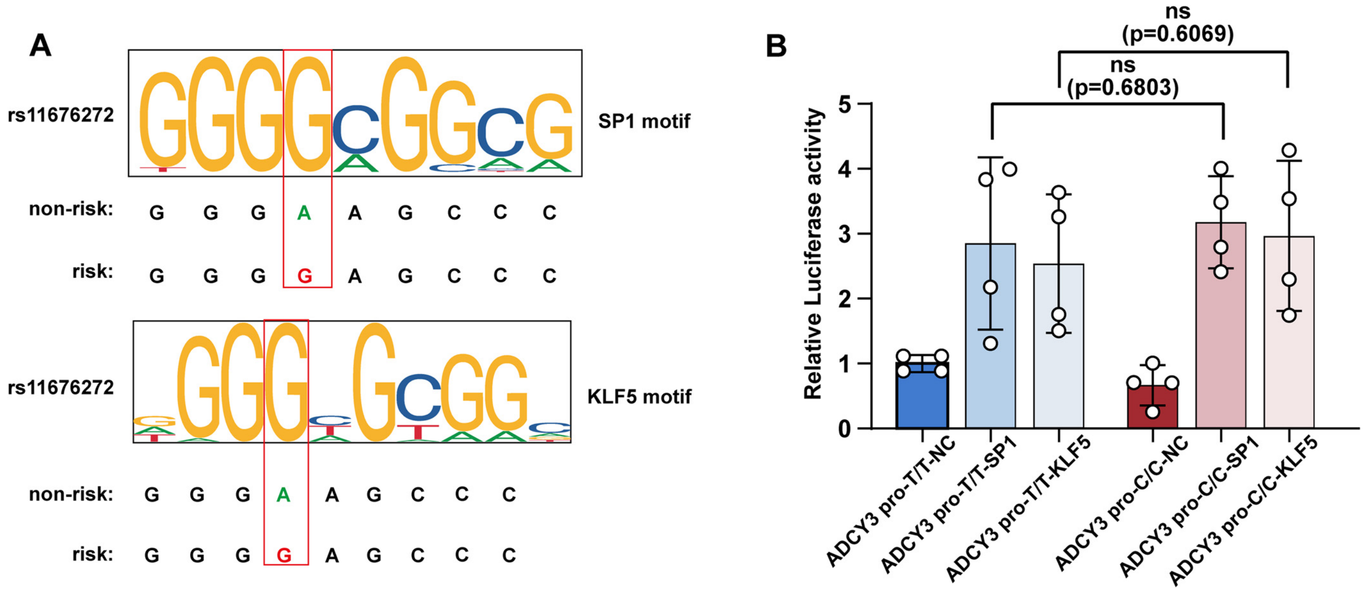

Figure EV3. rs11676272-T or C alleles do not affect the binding affinity of SP1 and KLF5.

(**A**) The SP1 binding motif (top) and KLF5 binding motif (bottom) and the rs11676272-C risk allele (complementary) and the rs11676272-A nonrisk allele (complementary) are shown. (**B**) Luciferase reporter assay performed after transient cotransfection of NIH3T3 cells with the indicated constructs. The DNA fragment containing either the T or C allele of the rs11676272 was cloned into the *ADCY3* promoter-driven luciferase reporter construct, which was cotransfected with either an empty vector or SP1 or KLF5-overexpressing vector. $n = 4$ groups from three independent experiments; All data were presented as mean ± SD, ns not significant by one-way ANOVA.

 © The Author(s)

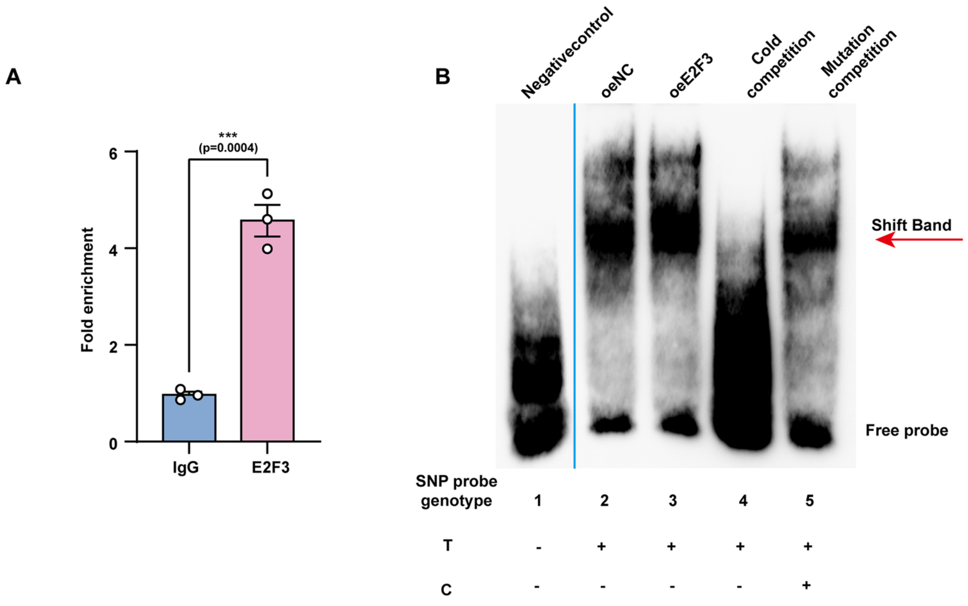

Figure EV4. rs11676272-C allele specifically disrupts E2F3 binding capacity, whereas the -T allele preserves robust E2F3 recruitment.

(**A**) ChIP-qPCR for *E2F3* at the DNA fragment containing rs11676272 region in HEK293T cells. $n = 3$ groups from three independent experiments. (**B**) EMSA showing differential affinities of the rs11676272-T nonrisk and -C risk alleles for E2F3. The red arrow indicates the shifted bands. Nuclear extracts were obtained from HEK293T cells transfected with either an empty vector or an E2F3-overexpressing construct. All data were presented as mean ± SD, $***p < 0.001$ by Student's *t*-test. Source data are available online for this figure.

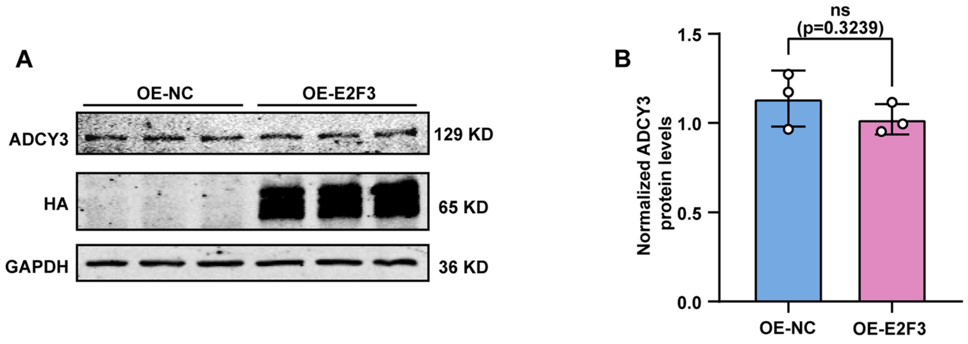

Figure EV5. E2F3 does not bind to the rs11676272-C risk allele and enhance the ADCY3 expression.

(**A**, **B**) Western blot showing E2F3 and ADCY3 expression levels in E2F3 overexpressing rs11676272-C/C cells. Cell lysates were obtained from HEK293T cells transfected with either an empty vector or an E2F3-overexpressing construct. GAPDH was used as a loading control. $n = 3$ groups from three independent experiments; All data were presented as mean ± SD, ns, not significant by Student's t-test.

 © The Author(s)

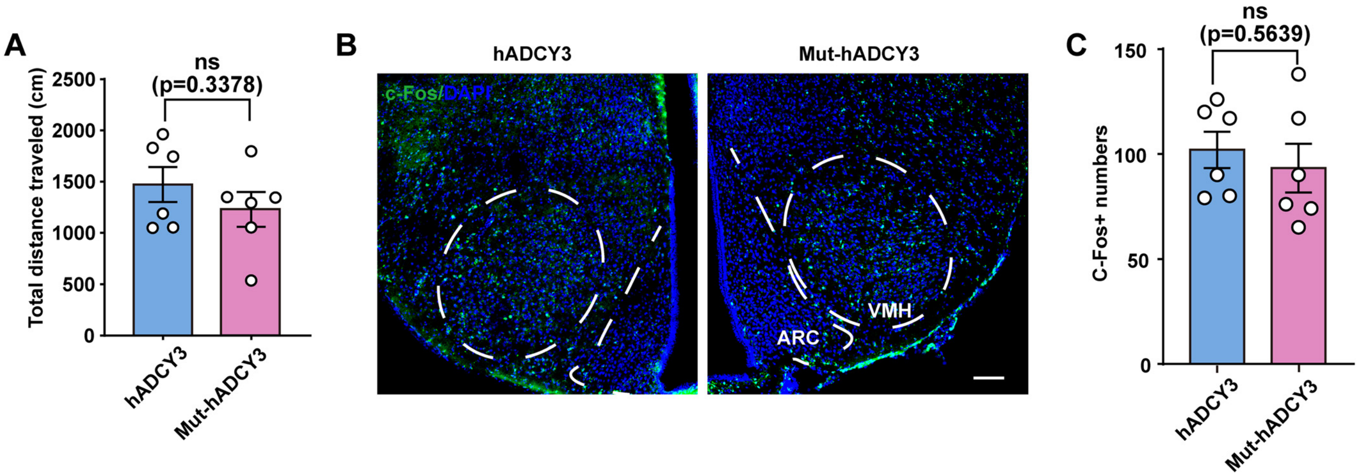

Figure EV6. The total locomotor activity and neuronal activation of hADCY3 and Mut-hADCY3 mice showed no significant differences.

(**A**) Total locomotor activity in hADCY3 and Mut-hADCY3 mice. $n = 6$ groups from three independent experiments. (**B**, **C**) Representative images (**B**) and quantification (**C**) of c-Fos immunofluorescence in VMH and ARC of the hADCY3 and Mut-hADCY3 mice. DAPI (blue) was used to stain nuclei. Scale bars, 100 μm. $n = 6$ groups from three independent experiments; All data were presented as mean ± SD; ns no significance by Student's *t*-test.

© The Author(s)

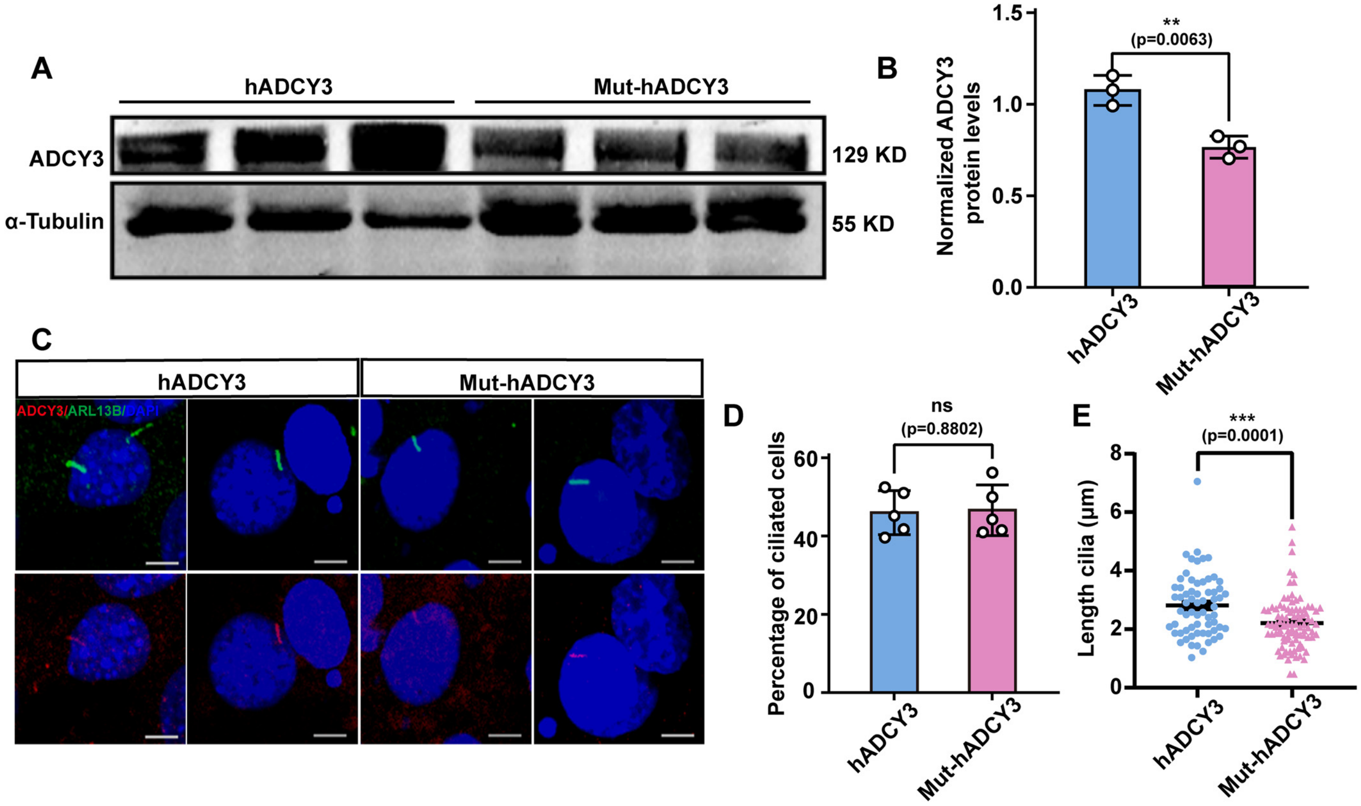

Figure EV7. The hADCY3 mice with the rs11676272-C risk variant result in lower ADCY3 levels and shorter cilia in the MEF cells.

(**A**, **B**) Western blot probing with anti-ADCY3 in MEF cells derived from hADCY3 and Mut-hADCY3 mice. α-Tubulin was used as a loading control. $n = 3$ groups from three independent experiments. (**C–E**) MEF cells from hADCY3 and Mut-hADCY3 mice were starved for 24 h, followed by staining with anti-ARL13B (green) and anti-ADCY3 (red) antibodies. DAPI (blue) was used to stain nuclei. Scale bars, 5 μm (**C**). Percentage of ciliated cells (**D**) and quantitative analysis of the cilium length (**E**) of MEF cells from hADCY3 and Mut-hADCY3 mice. $n = 3$ groups from three independent experiments; All data were presented as mean ± SD; $^{**}p < 0.01$, $^{***}p < 0.001$, ns no significance by Student's *t*-test.

 © The Author(s)

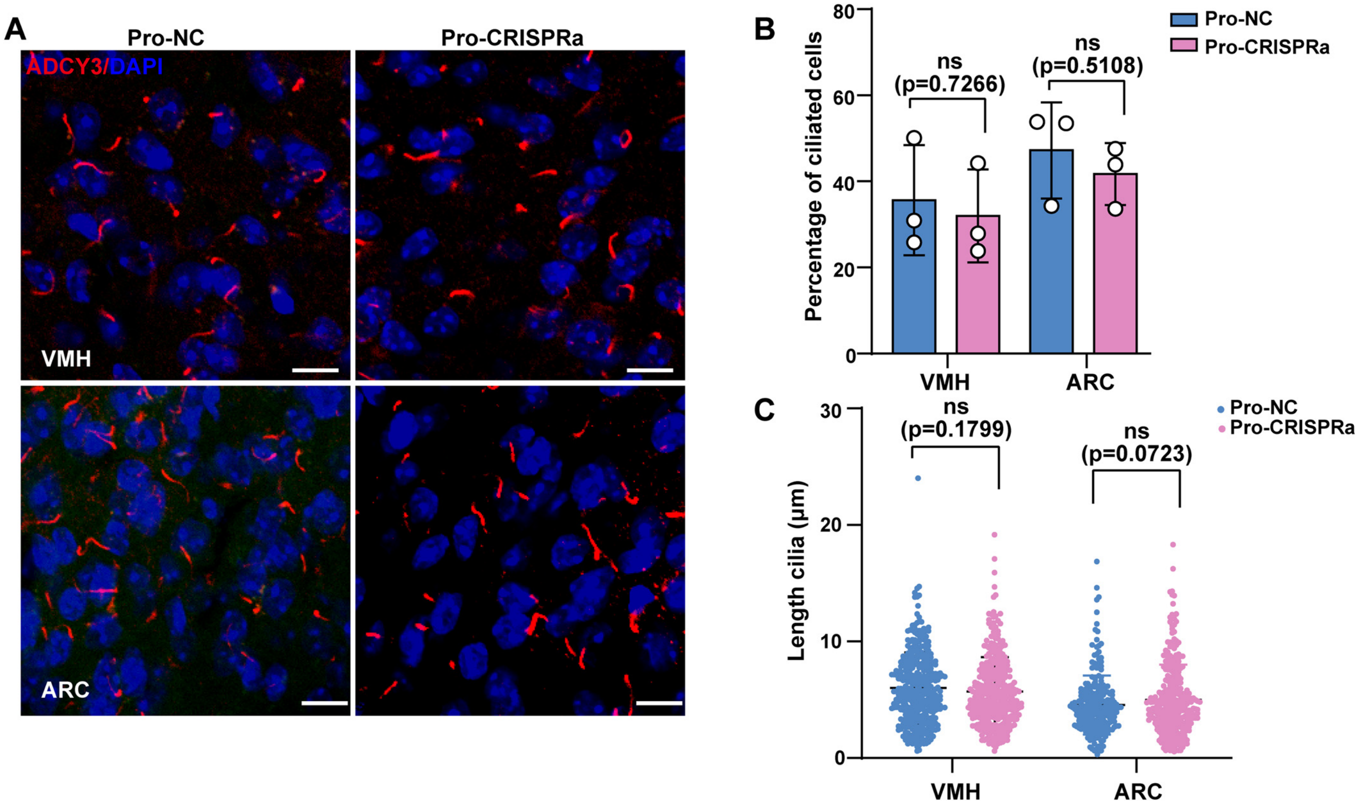

Figure EV8. CRISPRa-AAV injection in the VMH and ARC did not affect the length of cilia in wild-type mice.

(**A**) Cells in the VMH and ARC from the wild-type mice following injections of the pro-NC or pro-CRISPRa constructs were stained with anti-ADCY3 (red) antibody. DAPI (blue) was used to stain nuclei. Scale bars, 10 μm. (**B**, **C**) Percentage of ciliated cells in the VMH and ARC from the wild-type mice (**B**), and quantification of cilium length in the VMH and ARC from the wild-type mice (**C**). $n = 3$ groups from three independent experiments; The data were displayed as mean ± SD. ns no significance by one-way ANOVA.
